# Optimization design of car door structure considering aesthetics and safety

**DOI:** 10.1371/journal.pone.0357798

**Published:** 2026-09-28

**Authors:** Yu’an Ning, Kiesu Kim

**Affiliations:** College of Fine Arts, Silla University, Busan, Republic of Korea; Universiti Teknologi Malaysia - Main Campus Skudai: Universiti Teknologi Malaysia, MALAYSIA

## Abstract

To enhance the performance of multi-objective optimization algorithms in the optimization design of car door structures and comprehensively optimize the aesthetics and safety of car doors, an improved multi-objective grey wolf optimization algorithm is proposed. The core of this improvement lies not only in using the optimal solution set method for population initialization to enhance uniformity and diversity, but more importantly, in introducing a novel adaptive double population strategy. This strategy fundamentally reshapes the search behavior of the algorithm by dynamically balancing local development and global exploration throughout the optimization process. At the same time, a hierarchical optimization model for the car door structure is constructed, which organizes parameters on three functionally complementary and logically progressive levels: geometric layer, structural layer, and material layer. An aesthetic function is constructed using the curvature uniformity index, and a safety function is constructed combining collision intrusion and chest acceleration, forming a multi-objective joint evaluation function. The research results indicate that in the multi-objective testing function, the improved multi-objective grey wolf optimization algorithm has an inverse generation distance of 0.041, which is 32.7% lower than the original multi-objective grey wolf optimization algorithm. In the high-dimensional testing problem Door3, the inverted generational distance of the improved multi-objective grey wolf optimization algorithm is only 0.082, which is 58.6% higher than that of the non-dominated sorting genetic algorithm II. After improving the multi-objective grey wolf optimization algorithm, the aesthetic function value of car doors increases from 0.62 to 0.89, the collision intrusion decreases from 65 mm to 42 mm, and the chest acceleration decreases from 45g to 32g. This study provides an efficient algorithm and feasible solution for multi-objective optimization of car door structures, and customized designs can be obtained by adjusting the aesthetic and safety weights.

## 1. Introduction

The automobile industry has achieved vigorous development in recent years. As consumer living standards improve, people increasingly focus on vehicle aesthetics and safety [1]. As a key body component, the car door serves both as a passenger passage and a safety barrier, with its structural design directly affecting both aesthetics and safety [2]. Early door structures are relatively simple, mainly meeting basic functions with limited consideration of aesthetics and safety [3]. Currently, manufacturers have increased investment in door design to create products that are both beautiful and safe [4]. Some studies have focused on safety by optimizing anti-collision beams and reinforcement plates, while others have addressed aesthetics through exterior design and surface treatment. However, most research has concentrated on only one aspect, lacking in-depth exploration of the synergistic optimization of aesthetics and safety. Furthermore, existing studies have not fully considered consumers’ diverse needs in setting optimization goals and evaluation indicators [5].

Automotive design achieves a balance between lightweight and cost control through multi-material structural layout, topology optimization, and implicit parametric modeling, while meeting the stiffness and strength requirements under different operating conditions. X. Chen et al. combined topology optimization, entropy weight method, and TOPSIS for multi-material body lightweight design, achieving a 3.49% weight reduction and significant improvements in bending stiffness, torsional stiffness, and collision safety [6]. S. Li et al. developed an optimization framework integrating numerical simulation, material parameterization, surrogate models, and NSGA-II, reducing vehicle side mass by 9.21 kg with a lightweighting rate of 15.93% while maintaining collision resistance [7]. Q. Wang et al. addressed the stability of engine compartment doors under axial loads using graphene oxide reinforced materials and deep neural networks, demonstrating enhanced load-bearing capacity and deformation resistance [8]. Fang et al. proposed a multi-material structural design for steel car doors using aluminum and long fiber thermoplastics, achieving about 20% weight reduction while maintaining or improving mechanical performance [9]. P. Rostami et al. introduced a machine learning-based low-dimensional parameterization method for Multi-Objective Optimization (MOO) of engine hood inner panels, showing low computational complexity and improved accuracy and efficiency [10].

MOO is used to address optimization issues with multiple conflicting objectives, achieving balance and trade-off among multiple objectives by generating Pareto optimal solution sets. It is broadly utilized in engineering design, resource management, traffic optimization and other fields, which can effectively enhance the comprehensive performance of the system, reduce costs and achieve sustainability goals. P. Jangir et al. designed a multi-objective ocean predator algorithm grounded on elite non-dominated sorting and crowded distance mechanism for MOO problems, and analyzed the Pareto frontier of unconstrained, constrained, and engineering design problems with 32 different features. The results indicated that the performance of this algorithm was superior to comparative algorithms such as multi-objective water cycle algorithms [11]. X. Zhou et al. designed a combined optimization strategy for multi-objective collaborative design of rear seats in sedans, which includes a comprehensive genetic aggregation response surface surrogate model, NSGA-III algorithm, and multi-criteria decision-making method. The results indicated that optimizing the displacement of the rear seat headrest and backrest frame reduced material costs and weight by 7.1% and 5.54%, respectively [12]. S. Anosri et al. compared the performance of 12 metaheuristic algorithms on four drone design issues for the MOO problem of fixed wing unmanned aerial vehicle concept design. Research denoted that some algorithms had significant advantages and were effective in solving aircraft conceptual design problems [13].

However, the existing advanced MOO algorithms, such as NSGA-II, SPEA2, MOPSO, and MOGWO, still have the following deficiencies when dealing with complex engineering problems. First, the balance between convergence and diversity remains problematic. Traditional algorithms tend to converge prematurely and fall into local optima, while others like NSGA-II may suffer from slow convergence in later stages due to insufficient exploration [14,15]. Second, the complexity of problem modeling poses a major obstacle. Many engineering problems involve coupled parameters from multiple disciplines such as geometry, structure, and materials, yet traditional optimization models often flatten these dimensions, making it difficult to incorporate design logic and physical constraints [16,17]. Third, the quantification and integration of subjective performance indicators have long been a challenge. Soft indicators such as “aesthetics,” which rely on human visual cognition, are frequently excluded from optimization frameworks due to their subjectivity and measurement difficulty, leading to an imbalance between form and function in design outcomes [18,19]. The standard MOGWO exhibits two major limitations when applied to complex optimization problems:

(1) It tends to converge prematurely, that is, it gets trapped in the local Pareto optimal region before thoroughly exploring the target space.
(2) Its population diversity is often insufficient, especially in the later iterations, which limits the ability to obtain well-distributed non-dominated solution sets.

To overcome these shortcomings, the improved MOGWO integrates two key modifications:

(1) Initialization of the optimal solution set, that is, initializing the population using the low-difference point set (good point set) method instead of random initialization. This ensures from the very beginning that the distribution of individuals in the search space is more uniform and diverse, enhancing the algorithm’s early global exploration ability.
(2) Adaptive dual population strategy, that is, the population is dynamically divided into two subpopulations: one is led by the leading wolf (emphasizing local development), and the other is led by randomly selected wolves (emphasizing global exploration). The sizes of the two subpopulations are adaptively adjusted with the number of iterations, thereby achieving a continuous balance between dense local search and exploratory global search throughout the optimization process.

The innovation point of this study lies in:

(1) Structural redesign of MOGWO’s population dynamics. Improvement is not merely about parameter adjustment or minor expansion. It is a strategic reconstruction of the internal search mechanism of the algorithm. By combining the initialization of the optimal set with the adaptive dual population strategy, the basic search behavior of this algorithm is reshaped to systematically address its core weaknesses in convergence and diversity.
(2) Dynamic resource allocation between development and exploration. The adaptive dual population strategy introduces a new dynamic resource allocation mechanism, which can continuously manage the trade-off between local optimization and global exploration. This ensures that the algorithm gradually converges towards the high-quality Pareto frontier while maintaining high diversity.
(3) Enhanced robustness and performance – The collaborative combination of the two mechanisms not only mitigates premature convergence and increases the diversity of solutions, but also leads to significantly superior and more robust optimization performance, as verified by comprehensive tests on benchmark functions and real-world gate design problems.

The innovations of this study are: (1) The improved MOGWO algorithm is not a simple parameter modification of traditional MOGWO, but a structural reconstruction from the perspective of population dynamic behavior. Unlike existing variants that commonly use a fixed population strategy or single guidance mechanism, this paper designs an adaptive dual-population strategy. (2) The core of this strategy lies in dynamically dividing the population based on the iteration process: one subpopulation is guided by the leader wolf and mainly undertakes local development tasks, with its size gradually expanding to enhance search depth; the other subpopulation is guided by random grey wolves and focuses on global exploration, with its size gradually decreasing to retain global search ability in later stages. Through this dynamic adjustment mechanism, the algorithm achieves a continuous balance between local development and global exploration throughout optimization. (3) By combining the optimal point set initialization method, the distribution uniformity and diversity of the initial population are further improved, providing good starting conditions for the subsequent adaptive balancing mechanism. This improvement path of “initialization homogenization → population dynamic differentiation → search behavior synergy” constitutes the main technical feature distinguishing this study from existing MOGWO variants. The potential perspectives are: Firstly, the research method can improve the uniformity of population distribution in MOO processes, and achieve a dynamic balance between local exploitation and global exploration, thereby alleviating the problems of traditional algorithms easily falling into local optima and having insufficient convergence stability. Secondly, the constructed hierarchical parameter model can systematically describe the key design parameters of the car door, and combined with a multi-objective function based on the Curvature Uniformity Index (CUI) and weighted risk value, it can achieve quantitative coordination between aesthetics and safety in car door structure optimization. Notably, the validation scope of the research is currently limited to simulation optimization under standard multi-objective testing functions and specific door structure parameters.

The contribution of the research mainly lies in two aspects. At the level of MOO methodology, an improved MOGWO algorithm is proposed, which effectively alleviates the problems of traditional algorithms being prone to falling into local optima and unstable convergence when dealing with complex problems. In the field of automotive door engineering design and optimization, a layered optimization model that comprehensively considers aesthetics and safety is developed, providing a quantifiable new method for achieving collaborative optimization of door “form” and “function.”

## 2. Methodology

### 2.1. Multi-objective optimization algorithm design

In the optimization design of car door structures, the performance of MOO algorithms directly affects the quality of optimization results. Traditional GWOs are prone to getting stuck in local optima and insufficient population diversity when dealing with complex problems [20]. To address these issues, research was conducted to improve MOGWO by using the best point set method for population initialization and designing an adaptive dual population strategy. The traditional random initialization method may lead to uneven distribution of the population in the search space, thereby affecting the global exploration ability of the algorithm. A good point set is a set of points that can be evenly distributed in a unit hypercube, with good uniformity and bias properties, and can effectively improve the diversity of the population [21]. The schematic diagram of random initialization and optimal point set initialization is shown in Fig 1.

**Fig 1:**
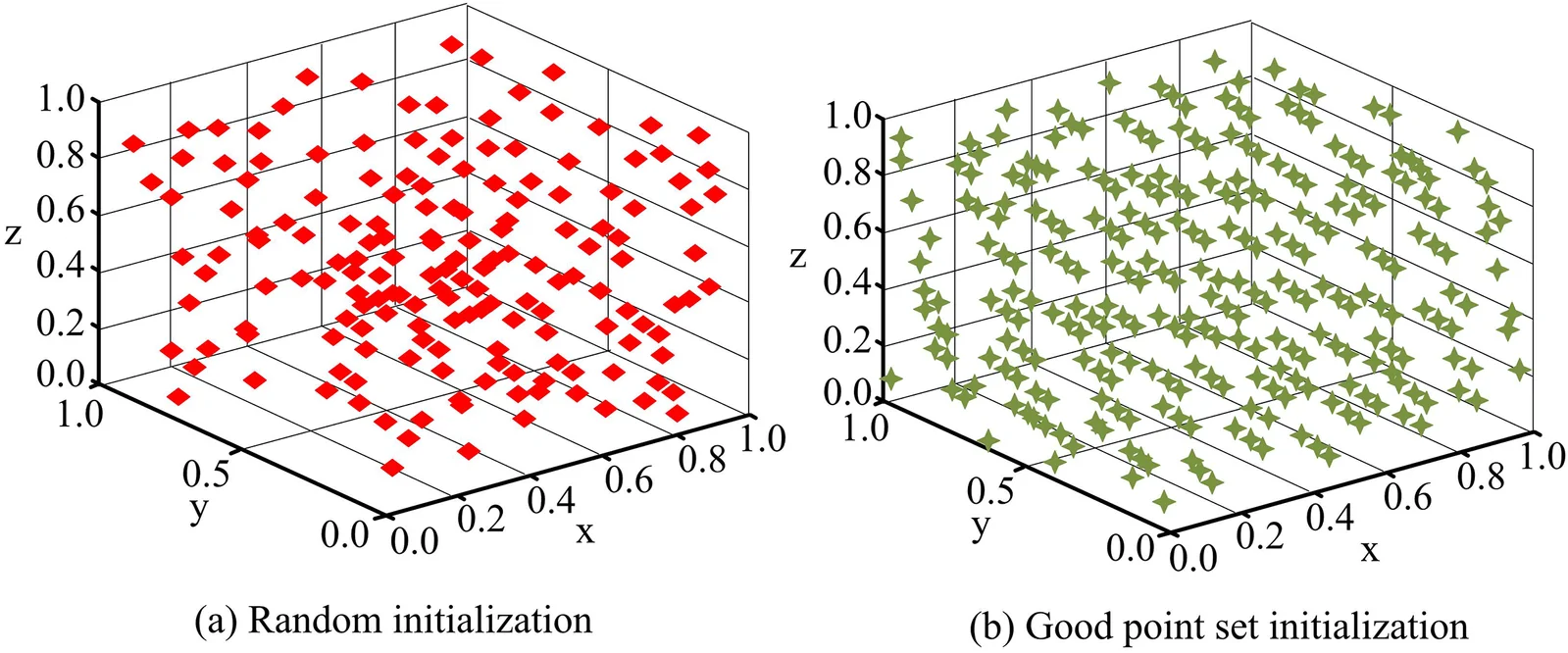
Schematic diagram of random initialization and optimal point set initialization.

Fig 1(a) shows a schematic diagram of random initialization, where individuals are unevenly distributed in the search space. Fig 1(b) shows the initialization of the optimal point set, where individuals are uniformly distributed within the domain. From this, the initialization of the optimal set improves the uniformity and diversity of the initial distribution of the population, which helps optimize the algorithm for better global exploration in the early stages. Assuming the population size is N and the dimension of the optimization problem is D, for the j th variable of the i th individual, the optimal point set method is used for initialization. The generation of the optimal point set is based on the optimal point theory in number theory. Selecting a generator g, the initial value $x_{i,j}^{\text{0}}$ of the i th individual in the j th dimension is calculated as shown in equation (1) [22].


$$\begin{array}{c} x_{i,j}^{\text{0}}=\frac{\left\lfloor i\cdot g^{i}\right\rfloor modN}{N}\times (u_{j}-l_{j})+l_{j} \end{array}$$ (1)

In equation (1), ⌊·⌋ represents the down rounding operation, $l_{j}$and $u_{j}$ indicate the lower and upper bounds of the j th dimensional variable, respectively. The optimal point set is mapped to the actual value range of the variable, so that the initial population is evenly distributed in the search space, providing rich initial information for optimizing the search [23]. To avoid the algorithm getting stuck in local optima and enhance its global exploration ability, an adaptive dual population strategy is studied and designed. This strategy divides the population into two subpopulations, guided by the leading wolf (i.e., the current optimal solution) and the random grey wolf, and the number of individuals in the subpopulations adaptively changes with the number of iterations. Assuming the current iteration count is t and the max iteration count is T, subpopulation 1 is guided by the leader wolf and mainly responsible for local development. Its individual number $N_{\text{1}}\left(t\right)$ gradually increases with the increase of iteration count to enhance the search for the current optimal region. Subpopulation 2 is guided by random grey wolves and is mainly responsible for global exploration. Its individual number $N_{\text{2}}\left(t\right)$ gradually decreases with the increase of iteration times to maintain a certain global search ability. The calculation of the number of individuals in the subpopulation is shown in equation (2).


$$\begin{array}{c} \left\{ \begin{array}{c} N_{\text{1}}\left(t\right)\text{=}\left\lfloor N\cdot {\left(\frac{t}{T}\right)}^{\text{2}}\right\rfloor +1\\ N_{\text{2}}\left(t\right)\text{=}N-N_{\text{1}}\left(t\right) \end{array}\right. \end{array}$$ (2)

In subpopulation 1, the leader wolf guides individuals to update their positions according to traditional grey wolf optimization algorithms, that is, by mimicking the social hierarchy and hunting behavior of grey wolves for position updates [24]. For the k th individual, the position update formula is denoted in equation (3).


$$\begin{array}{c} {\vec{x}}_{k}^{t+1}={\vec{x}}_{\alpha }^{t}-\vec{A}\cdot \left|\vec{C}\cdot {\vec{x}}_{\alpha }^{t}-{\vec{x}}_{k}^{t}\right| \end{array}$$ (3)

In equation (3), ${\vec{x}}_{\alpha }^{t}$ means the position vector of the t th generation leader wolf, and $\vec{A}$ and $\vec{C}$ denote coefficient vectors. The formula for calculating the coefficient vector is shown in equation (4).


$$\begin{array}{c} \left\{ \begin{array}{c} \vec{A}=2\vec{a}\cdot {\vec{r}}_{\text{1}}-\vec{a}\\ \vec{C}=2\cdot {\vec{r}}_{\text{2}} \end{array}\right. \end{array}$$ (4)

In equation (4), $\vec{a}$ represents a linear decrease from 2 to 0 with the number of iterations, and ${\vec{r}}_{\text{1}}$ and ${\vec{r}}_{\text{2}}$ denote random vectors within the interval [0,1]. In subpopulation 2, the random grey wolf randomly selects an individual from the current population as a guide to guide the individuals in subpopulation 2 to update their positions, to increase the randomness and diversity of the search. If the randomly selected guide position is ${\vec{x}}_{\text{rand}}^{t}$, the formula for updating the position of the m th individual is denoted in equation (5) [25].


$$\begin{array}{c} {\vec{x}}_{m}^{t+1}={\vec{x}}_{\text{rand}}^{t}+\vec{\beta }\left|\vec{\gamma }\cdot {\vec{x}}_{\text{rand}}^{t}-{\vec{x}}_{m}^{t}\right| \end{array}$$ (5)

In equation (5), $\vec{\beta }$ and $\vec{\gamma }$ represent random coefficient vectors with values ranging from 0 to 1. The flowchart of the adaptive dual population strategy is shown in Fig 2.

**Fig 2:**
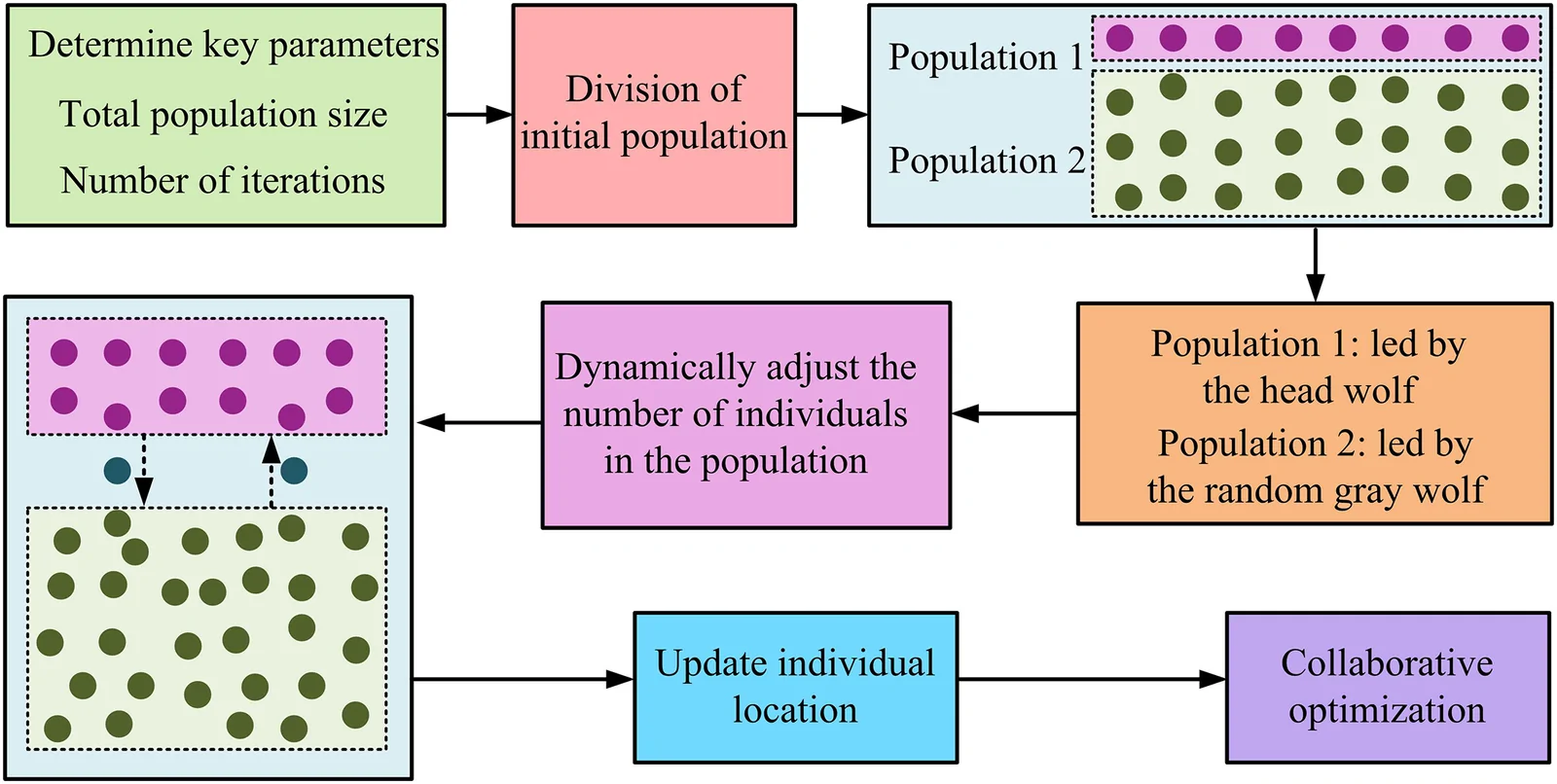
Flow chart of adaptive dual population strategy.

In Fig 2, parameters such as the total population size and iteration count are first determined, and then the population is divided into two subpopulations. Subpopulation 1 is guided by the leading wolf and focuses on local development. Its individual number gradually increases with the number of iterations, which can enhance the search for the current optimal area. Subpopulation 2 is guided by random grey wolves and is mainly responsible for global exploration. Its number of individuals gradually decreases with the increase of iteration times to maintain a certain level of global search ability. During the iteration, the number of individuals in the two subpopulations is adaptively adjusted according to the set formula, and the positions of individuals in the two subpopulations are updated according to corresponding rules. Finally, through this dynamic adjustment and collaborative search, the overall performance of the algorithm is improved. The improved MOGWO algorithm process is shown in Fig 3.

**Fig 3:**
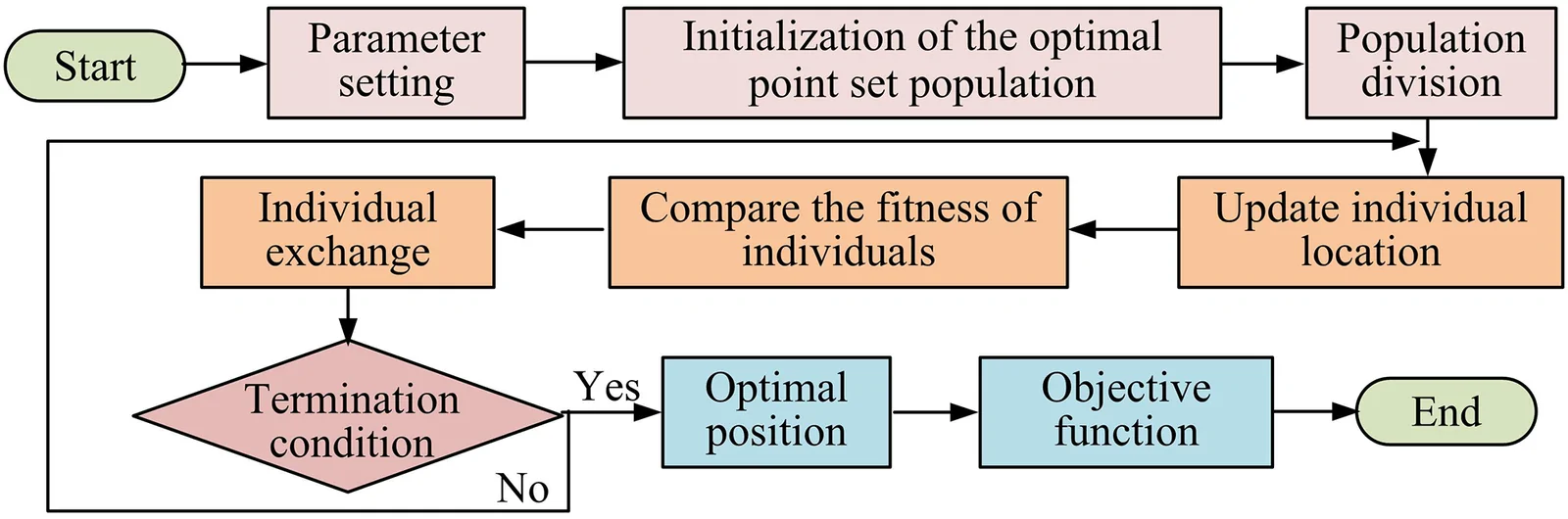
Improved MOGWO algorithm process.

As shown in Fig 3, after the algorithm starts, it first sets the parameters and then initializes the population using the optimal set method. Next, the population is divided, and the iterative optimization phase begins, updating the individual positions according to the position update formula. In each iteration, the fitness of individuals in the two populations is compared. If individuals in the two populations have complementary advantages on a specific objective, then individuals are swapped. When the preset max number of iterations is reached or the termination conditions such as convergence accuracy are met, the optimal position is output, the objective function is calculated, and the algorithm is terminated.

### 2.2. Design of optimization model for car door structure

The optimization of car door structure involves multiple levels of parameters. To clearly describe and process these parameters, a hierarchical modeling method is adopted to divide the parameter space into geometric layer, structural layer, and material layer. The hierarchical model diagram of parameter space is shown in Fig 4.

**Fig 4:**
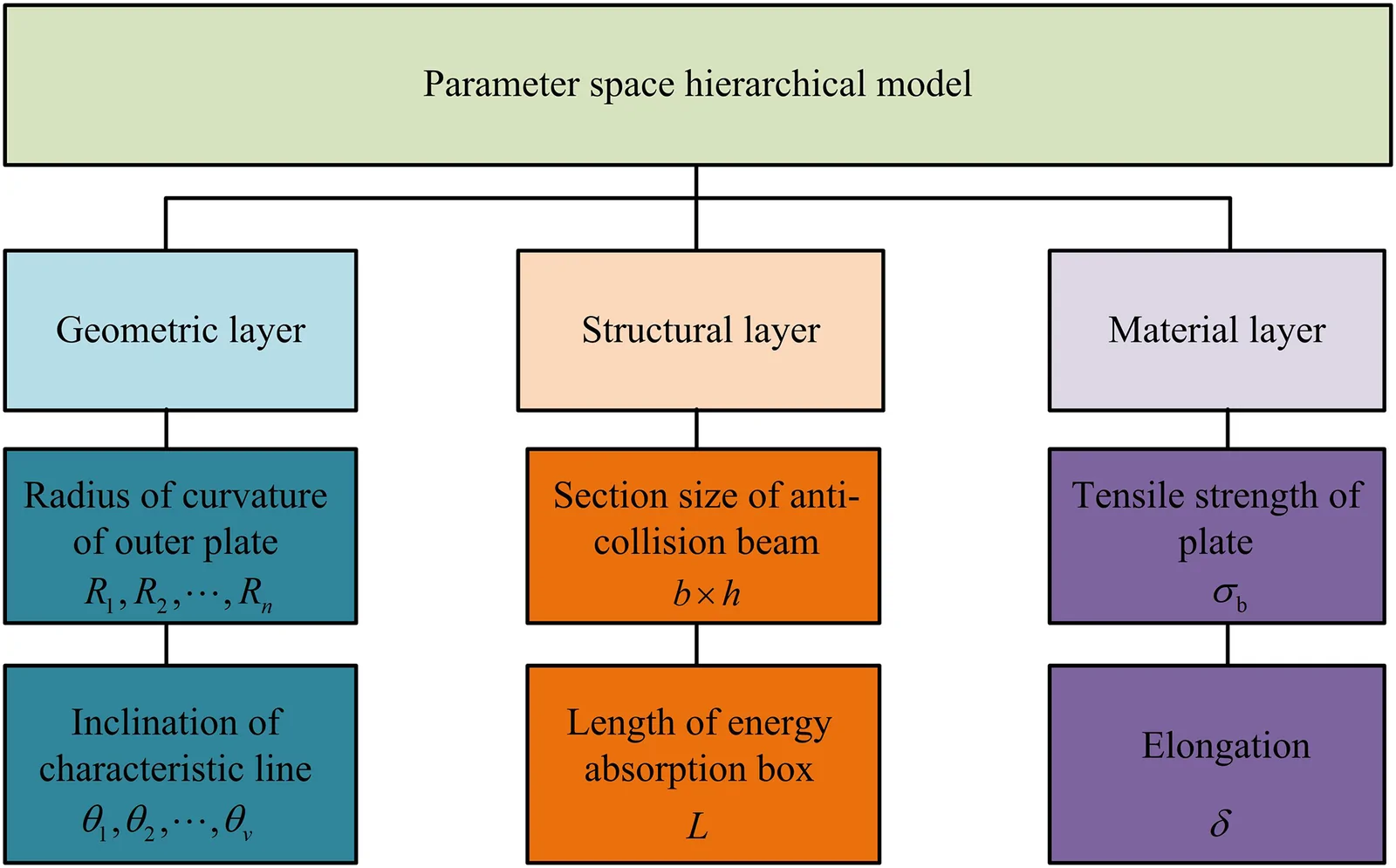
Hierarchical model of parameter pace.

In Fig 4, the geometric layer mainly focuses on the external shape and geometric features of the car door, which directly affect the aesthetics of the car door. The specific parameters include the curvature radius $R_{\text{1}},R_{\text{2}},\cdots ,R_{n}$ of the outer panel and the inclination angles $\theta_{\text{1}},\theta_{\text{2}},\cdots ,\theta_{v}$ of the characteristic line. The curvature radius $R_{\text{1}},R_{\text{2}},\cdots ,R_{n}$ of the outer panel represent the curvature magnitude at different positions on the door outer panel. The larger the curvature radius, the smoother the surface, and vice versa, the more curved it is [26]. The feature line inclination angles $\theta_{\text{1}},\theta_{\text{2}},\cdots ,\theta_{v}$ refer to the angles between the feature line on the car door and the horizontal direction, which is used to describe the direction and inclination degree of the feature line and has a significant impact on the overall visual effect of the car door. The structural layer parameters mainly involve the internal structure of the car door, which directly affects the safety of the car door. The parameters include the cross-sectional dimensions b×h of the anti-collision beam and the length L of the energy absorbing box. The width b and height h of the anti-collision beam section determine its bending and torsional stiffness, affecting its load-bearing capacity and deformation mode during collision. The length L of the energy absorbing box affects its energy absorption efficiency. A longer energy absorbing box can absorb more energy through greater deformation during a collision, thereby reducing the impact on the passenger compartment [27]. The research mainly considers the side collision condition. In this operating condition, the car door, as the main structural component on the side of the vehicle, directly bears the collision load and affects the living space of passengers. The collision intrusion amount can directly reflect the degree of intrusion of the structure into the living space. The chest acceleration is used to evaluate the protective effect of the restraint system and structural energy absorption on the chest of passengers. The material layer parameters mainly consider the mechanical properties of the sheet, including the tensile strength $\sigma_{\text{b}}$ and elongation δ of the sheet. The tensile strength $\sigma_{\text{b}}$ denotes the ability of the material to resist tensile fracture, and the elongation δ means the plastic deformation ability of the material before fracture. Different combinations of tensile strength and elongation will affect the strength, stiffness, and energy absorption performance of car doors [28].

To comprehensively consider the aesthetics and safety of car doors, a joint evaluation function is developed, including aesthetics function, safety function, and multi-objective function. In the design of automobile body shape, the surface quality of the outer covering directly affects the visual aesthetics. The Curvature Uniformity Index (CUI) is extracted from a discrete point mesh uniformly distributed on the surface of the exterior panel of the car door, and the sampling density is determined by the resolution of the surface mesh in the finite element model. To ensure comparability between different design schemes, the original curvature values of each sampling point are normalized based on the maximum absolute curvature observed at all sampling points within the same design, resulting in a dimensionless index. CUI is then calculated as the ratio of the mean normalized curvature value to the sum of the mean and standard deviation, which constrains the index within the [0,1] interval. This indicator reflects the smoothness and continuity of surface transitions. A higher curvature uniformity indicates smaller local fluctuations in surface curvature, which is associated with smoother light and shadow reflection effects. The use of CUI as an aesthetic proxy indicator is in line with the common practice of evaluating the quality of automotive styling surfaces, and curvature continuity is considered one of the primary indicators of visual quality in this field. The calculation formula is shown in equation (6).


$$\begin{array}{c} CUI=1-\frac{\sigma_{\text{k}}}{\overset{―}{k}+\varepsilon } \end{array}$$ (6)

In equation (6), CUI represents the CUI, and $\overset{―}{k}$ and $\sigma_{\text{k}}$ represent the average and standard deviations of the curvature values of each discrete point on the surface of the door outer panel, respectively. ε represents a very small positive number used to avoid situations where the denominator is zero. The range of CUI values is [0,1], with larger values indicating better curvature uniformity and higher aesthetic appeal. The safety function considers the collision intrusion I and chest acceleration a during the collision, and evaluates the safety of the car door by calculating their weighted risk values. The collision intrusion amount I refers to the distance that the car door intrudes into the passenger compartment during the collision process. The larger the intrusion amount, the greater the threat to the safety of the passengers [29]. The chest acceleration a reflects the acceleration impact on the passenger’s chest during the collision process, and excessive chest acceleration can cause injury to the passenger’s chest. Assuming the weight of collision intrusion is $w_{\text{I}}$, the weight of chest acceleration is $w_{\text{a}}$, and $w_{\text{I}}+w_{\text{a}}\text{=}1$. Based on safety standards and actual situations, $w_{\text{I}}\text{=}0.6$ and $w_{\text{a}}\text{=}0.4$ are taken. The formula for calculating the safety function S is denoted in equation (7).


$$\begin{array}{c} S=w_{\text{I}}\cdot \frac{I-I_{min}}{I_{max}-I_{min}}+w_{\text{a}}\cdot \frac{a-a_{min}}{a_{max}-a_{min}} \end{array}$$ (7)

In equation (7), $I_{min}$ and $I_{max}$ respectively indicate the mini and max allowable values of collision intrusion, while $a_{min}$ and $a_{max}$ respectively represent the mini and max allowable values of chest acceleration. This function normalizes two indicators and weights them together, with smaller values indicating higher security [30]. The multi-objective function combines the aesthetics function and safety function to optimize both the aesthetics and safety of the car door. Assuming the weight of the aesthetic function is $w_{\text{1}}$, the weight of the safety function is $w_{\text{2}}$, and $w_{\text{1}}+w_{\text{2}}=1$. Based on safety standards and actual situations, $w_{\text{1}}\text{=}0.4$ and $w_{\text{2}}\text{=}0.6$ are taken. The calculation formula for the multi-objective function F is denoted in equation (8).


$$\begin{array}{c} F=w_{\text{1}}\cdot CUI+w_{\text{2}}\cdot \left(1-S\right) \end{array}$$ (8)

In equation (8), (1−S) converts the safety function into a higher value for higher safety, to unify the optimization direction with the aesthetics function, that is, to maximize the multi-objective function F. The process of applying the improved MOGWO algorithm to the optimization model of car door structure is shown in Fig 5.

**Fig 5:**
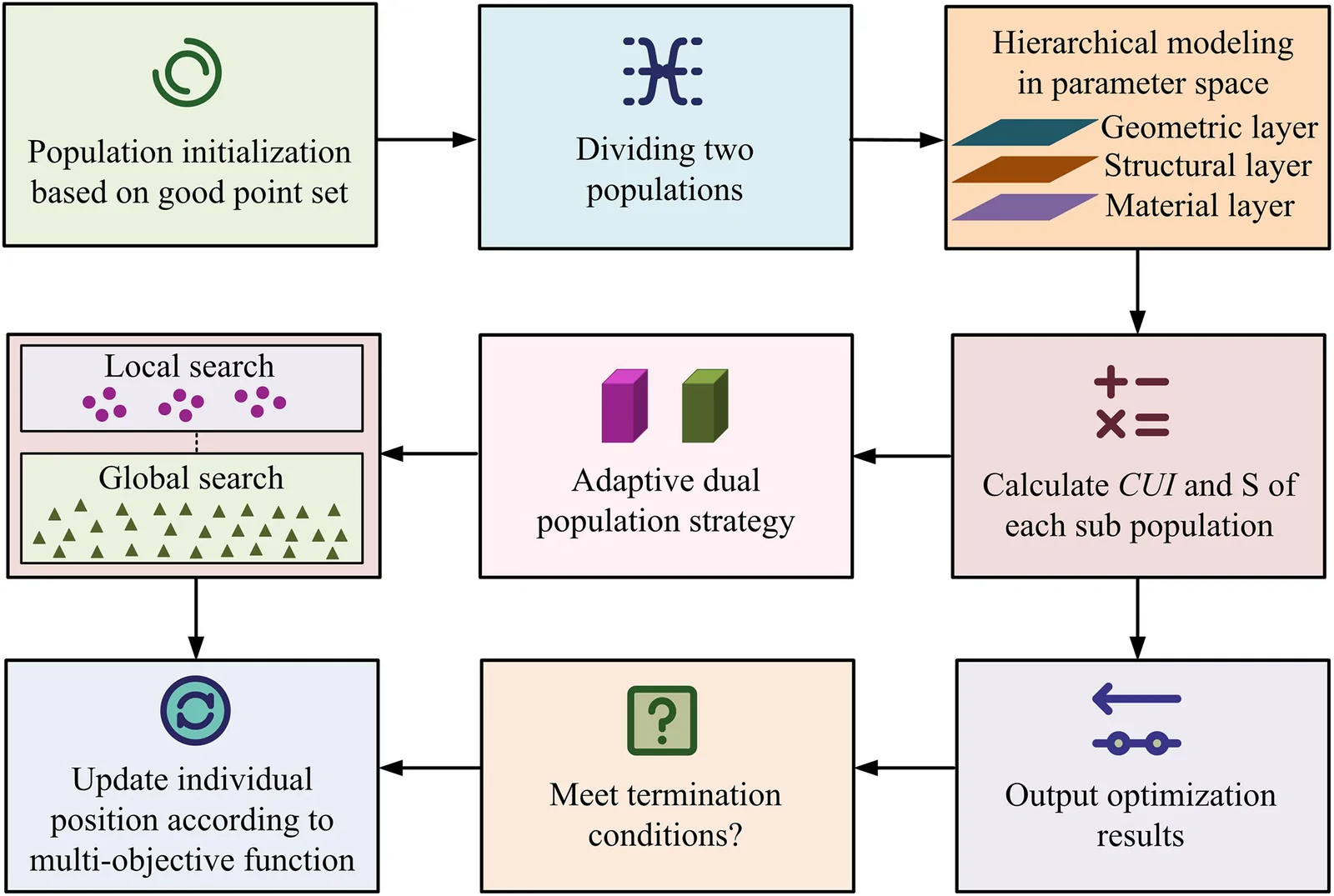
Process of applying the improved MOGWO algorithm to the optimization model of car door structure (Icon source: https://iconpark.oceanengine.com/official).

In Fig 5, the optimal point set method is first used to initialize the population. Subsequently, a layered modeling of the parameter space of car doors is carried out, clarifying the parameter dimensions of the geometric layer (outer panel curvature, characteristic line inclination), structural layer (collision beam size, energy absorbing box length), and material layer (matching of plate tensile strength and elongation). Next is to calculate the geometric, structural, and material parameters of each individual in the population. Based on these parameters, it first calculates the aesthetic function through curvature changes to measure the smoothness of the car door appearance. Combining the collision intrusion amount and chest acceleration weighted risk, the safety function is calculated, and the two together form a multi-objective function. Afterwards, the adaptive dual population strategy is initiated to dynamically partition subpopulations guided by the head wolf (local search) and random gray wolf (global exploration), adjust the subpopulation size iteratively, and update individual positions. Finally, determine whether the iteration termination condition is met, and if so, output the optimal solution for the car door structure. If not met, return to calculate individual parameters and continue iterative optimization until the design goal is achieved. Among the geometric layer parameters, the range of values for the curvature radius of the outer panel covers surface shapes from relatively flat to significantly curved, and the range of values for the inclination angle of the characteristic line covers typical angles in common car door designs. In the structural layer parameters, the section width and height of the anti-collision beam, as well as the length of the energy absorbing box, are set with upper and lower limits based on the constraints of the typical interior space of the car door and the feasibility of the manufacturing process. In the material layer parameters, the tensile strength and elongation at break of the sheet metal are set within the range of values based on the commonly used automotive stamping steel plate material performance spectrum. The normalized extreme values of collision intrusion and chest acceleration in the safety function are determined based on the performance limits specified in the current side collision safety regulations. The two indicators are linearly normalized and summed with fixed weights. The default values for aesthetic weight and safety weight in the multi-objective function are both set to 0.5 and can be adjusted according to design requirements. The optimization algorithm and finite element simulation are coupled offline: in each iteration step, the algorithm generates design variables and writes them to an intermediate file. The finite element model reads the file and performs collision simulation, and then returns the calculation results to the optimization algorithm. Each evaluation independently calls the simulation once.

## 3. Results

### 3.1. Experimental environment and parameter settings

The experiment was carried out on a high-performance computing cluster, and the experimental environment configuration is shown in Table 1. In terms of hardware, it adopted a 64 core Intel Xeon Platinum 8358 processor, paired with 256GB DDR4 memory. The graphics card used NVIDIA A100 GPU and supported parallel computing acceleration. The software environment was developed based on Python 3.9, using PyGMO 2.19 for algorithm implementation, and MATLAB R2023b for data visualization and analysis.

**Table 1:** Experimental environment configuration.

Environment	Configuration	Parameter
Hardware	Processor	Intel Xeon Platinum 8358 (64 cores, 2.6GHz)
	Memory	256GB DDR4
	Graphics card	NVIDIA A100 (40GB video memory)
	Storage	2TB NVMe SSD
Software	Operating system	Ubuntu 20.04 LTS
	Development language	Python 3.9 + MATLAB R2023b
	Algorithm library	PyGMO 2.19

Four sets of multi-objective test functions (ZDT1-ZDT4) and three sets of high-dimensional test problems related to custom door optimization (Door1-Door3) were set up in the experiment to evaluate the algorithm performance. Comparative algorithms included GWO, MOGWO, NSGA-II, and SPEA2. The core parameter settings of the algorithm are shown in Table 2, where the optimal point set generator used a golden ratio constant of 0.618, the initial ratio of the two populations was 1:1, and the adaptive coefficients α and β controlled the rate of change in the sub population size, respectively. The population size was set to 200 and the maximum number of iterations was set to 500. The population size can ensure sufficient coverage of the algorithm in the parameter space while balancing computational costs and search capabilities. The optimal set generator adopted a golden ratio constant of 0.618, which can make the distribution deviation of the initial points in the unit hypercube reach the theoretical lower bound. In the adaptive dual population strategy, the adaptive coefficient for controlling the growth rate of subpopulation 1 was set to 0.8, and the adaptive coefficient for controlling the decay rate of subpopulation 2 was set to 0.9, to ensure smooth changes in subpopulation size throughout the entire iteration process.

**Table 2:** Core parameter settings for algorithms.

Parameter	Value	Explanation
Population size	200	Total number of individuals
Maximum number of iterations	500	Iteration termination condition
Good point set generator	0.618	The generation parameter of the optimal point set in number theory
The initial proportion of subpopulation 1	0.5	The initial proportion of the sub-population led by the head Wolf
The initial proportion of subpopulation 2	0.5	The initial proportion of the random gray Wolf guide subpopulation
Adaptive coefficient	0.8	The growth rate of subpopulation 1 size
Adaptive coefficient	0.9	The rate of size decay of subpopulation 2
Compare algorithm parameters	All adopted the optimal parameters recommended in the literature	/

The finite element simulation is conducted using the LS-DYNA explicit solver and employs double precision calculations. The car door assembly includes an outer panel, an inner panel, a collision beam, and an energy absorbing box. It is discretized using four node Belytschko TSay shell elements, with a characteristic element size of 5 mm. The collision beam and its mounting bracket area adopt a 2 mm refined grid. The grid quality control adopts the standard of Jacobian ratio higher than 0.7 and aspect ratio lower than 5:1. The steel plate material is described using a segmented linear plastic material model combined with Cowper Symonds strain rate sensitivity. The benchmark design adopts a tensile strength of 400 MPa and an elongation of 20%, while the optimized design adopts a tensile strength of 500 MPa and an elongation of 25%. Both have an elastic modulus of 210 GPa and a density of 7.85 g/cm3. Automatic single-sided contact is defined for potential self contact within the deformed car door structure, and automatic surface contact is defined between the car door and the side collision barrier, as well as between the car door and the surrounding white body structure. The static friction coefficient of all contact pairs is taken as 0.15, and the dynamic friction coefficient is taken as 0.10. The side impact condition is simulated according to the US Federal Motor Vehicle Safety Standard FMVSS 214, which requires a movable deformable barrier with a mass of 1368 kg to impact the vehicle perpendicular to the longitudinal axis direction at a speed of 54 km/h. The deformable barrier panel adopts an aluminum honeycomb structure with a crush strength of 0.69 MPa. The passenger response was evaluated using the ES-2re side impact dummy model representing the 50th percentile adult male, with chest acceleration recorded from the chest acceleration sensor. The simulation termination time is set to 120 ms to cover the peak intrusion and subsequent rebound stages. The intrusion amount was measured at 12 monitoring nodes located in the vicinity of the door inner panel and B-pillar.

### 3.2. Performance testing of multi-objective optimization algorithm

The comparison chart of Pareto front of different algorithms under multi-objective testing function is shown in Fig 6. Figs 6 (a)-6 (d) show the comparison of the accumulated front of ZDT1-ZDT4, respectively. The horizontal axis corresponds to objective 1 and objective 2, and the improved MOGWO (blue dots) produced a more uniform distribution of Pareto front compared to other algorithms, with a wider distribution range on both objectives. From this, the improved MOGWO exhibits excellent performance in finding well distributed and diverse optimal trade-off solution sets.

**Fig 6:**
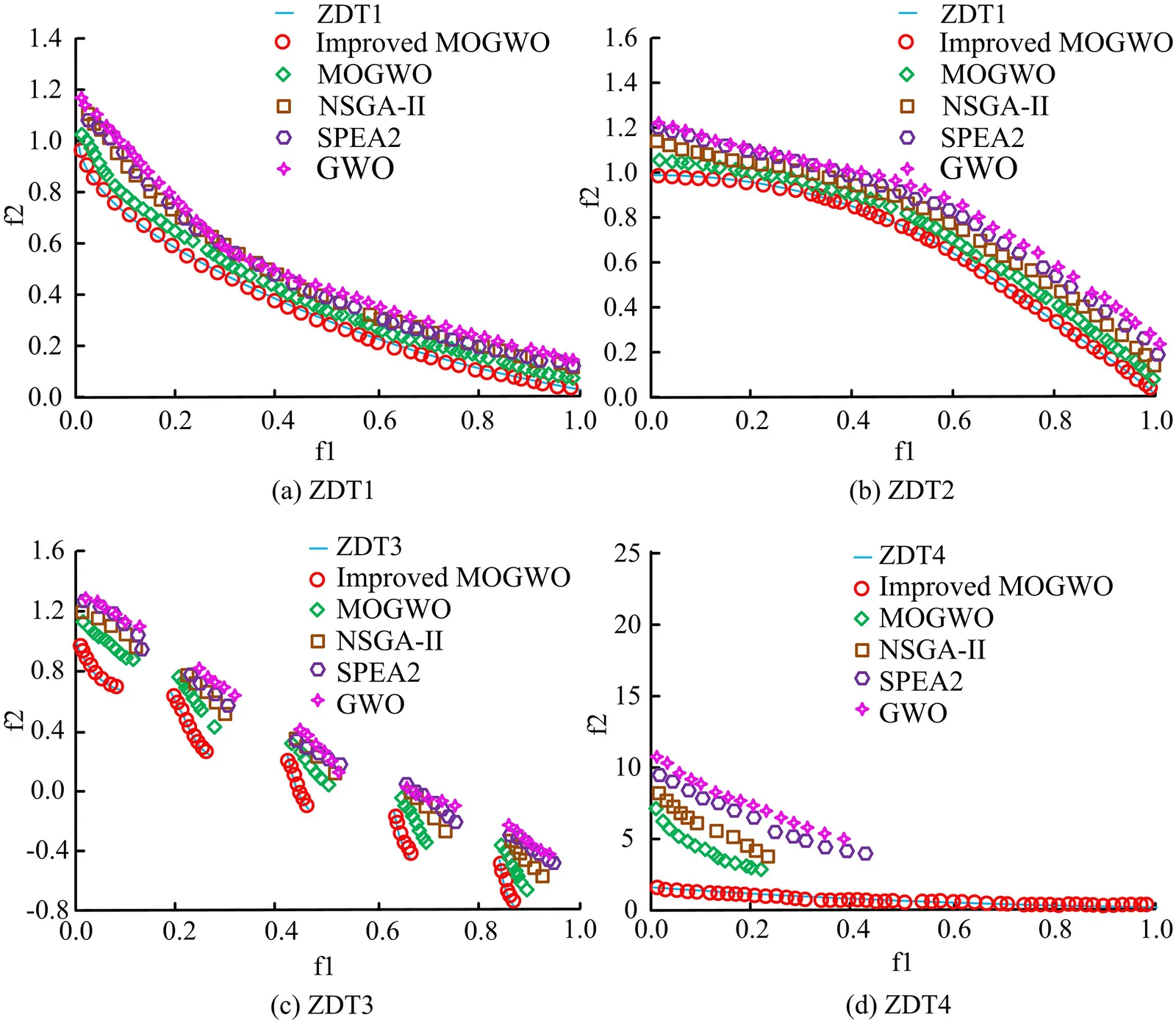
Comparison of Pareto front of different algorithms under multi-objective testing function.

The IGD convergence curves of different algorithms under multi-objective testing functions are shown in Fig 7. The horizontal and vertical axes represent the number of iterations and IGD values, respectively. The improved MOGWO curve (blue) decreased more rapidly and could stabilize at a lower value earlier (about 100 iterations), while other algorithms had a smoother decrease and experienced fluctuations in the later stages. The improved MOGWO could converge to a higher quality Pareto front faster and more stably. In the ZDT1-ZDT4 test function, the curve descent of traditional MOGWO and NSGA-II comparison algorithms was relatively gentle, and there was still some fluctuation in the later stage of iteration, resulting in a slower convergence speed. This indicates that the improved MOGWO algorithm, through optimal point set initialization and adaptive dual population strategy, can more efficiently explore the search space, approach the optimal solution faster, and significantly outperform the comparative algorithm in terms of iteration convergence times and convergence stability.

**Fig 7:**
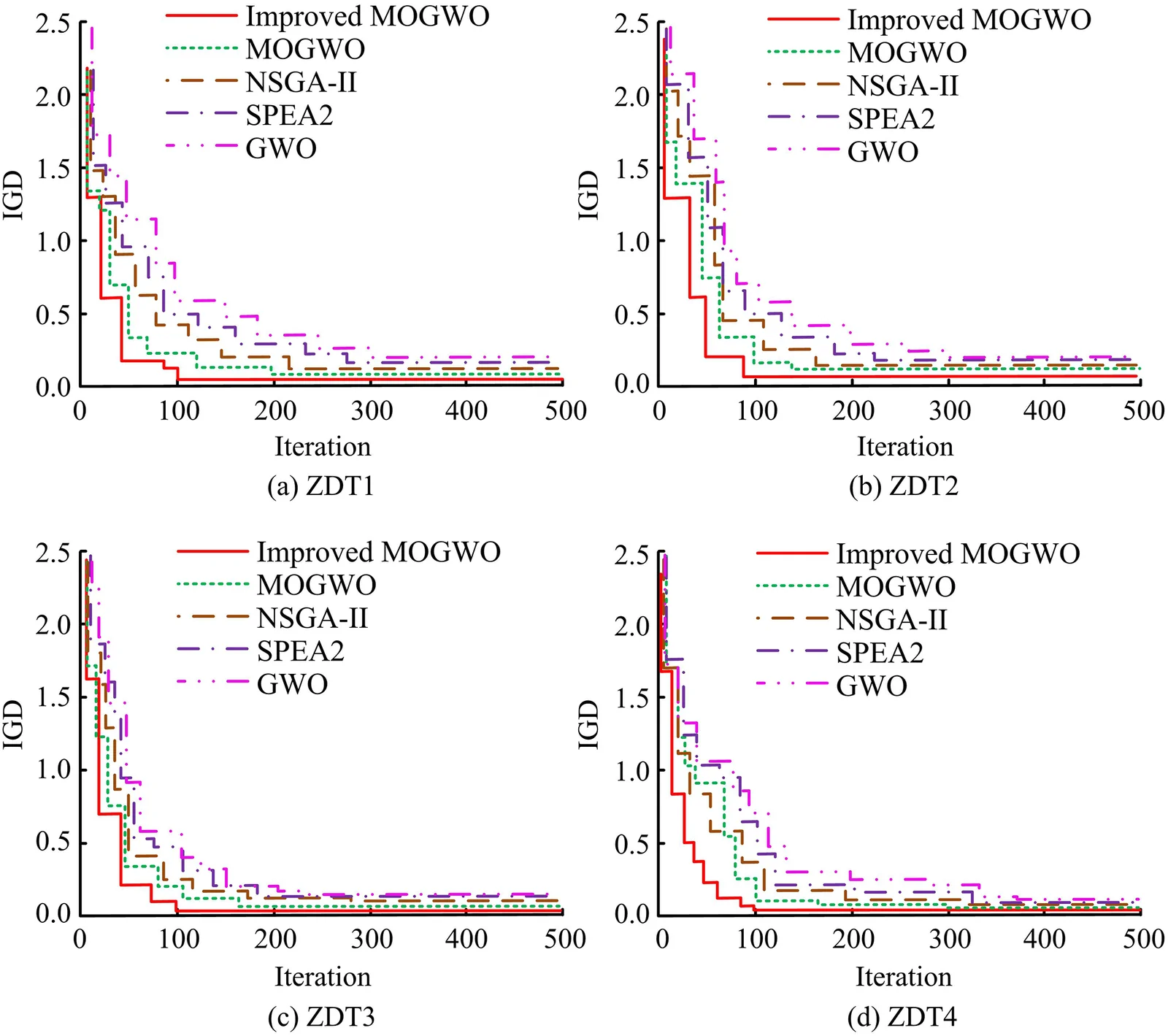
IGD convergence curves of different algorithms under multi-objective testing function.

The comparison of IGD and Hypervolume indicators of different algorithms under multi-objective testing functions is shown in Fig 8. The horizontal axis and vertical axis represent the test function and IGD value, respectively. The improved MOGWO consistently achieved the lowest IGD value and the highest Hypervolume value on all test functions. From this, the proposed algorithm outperforms the compared algorithms in terms of convergence and solution quality/diversity. In Fig 8(a), in the ZDT1-ZDT4 test function, the IGD values of the improved MOGWO were significantly lower than those of the comparison algorithms. The improved MOGWO on ZDT2 had an IGD of 0.041, which was 32.7% lower than the original MOGWO and a standard deviation reduction of 41.2%. In Fig 8(b), the improved MOGWO showed significantly higher Hypervolume values than the comparison algorithm on all test functions, with a value of 0.812 on ZDT4, which was 24.5% higher than SPEA2.

**Fig 8:**
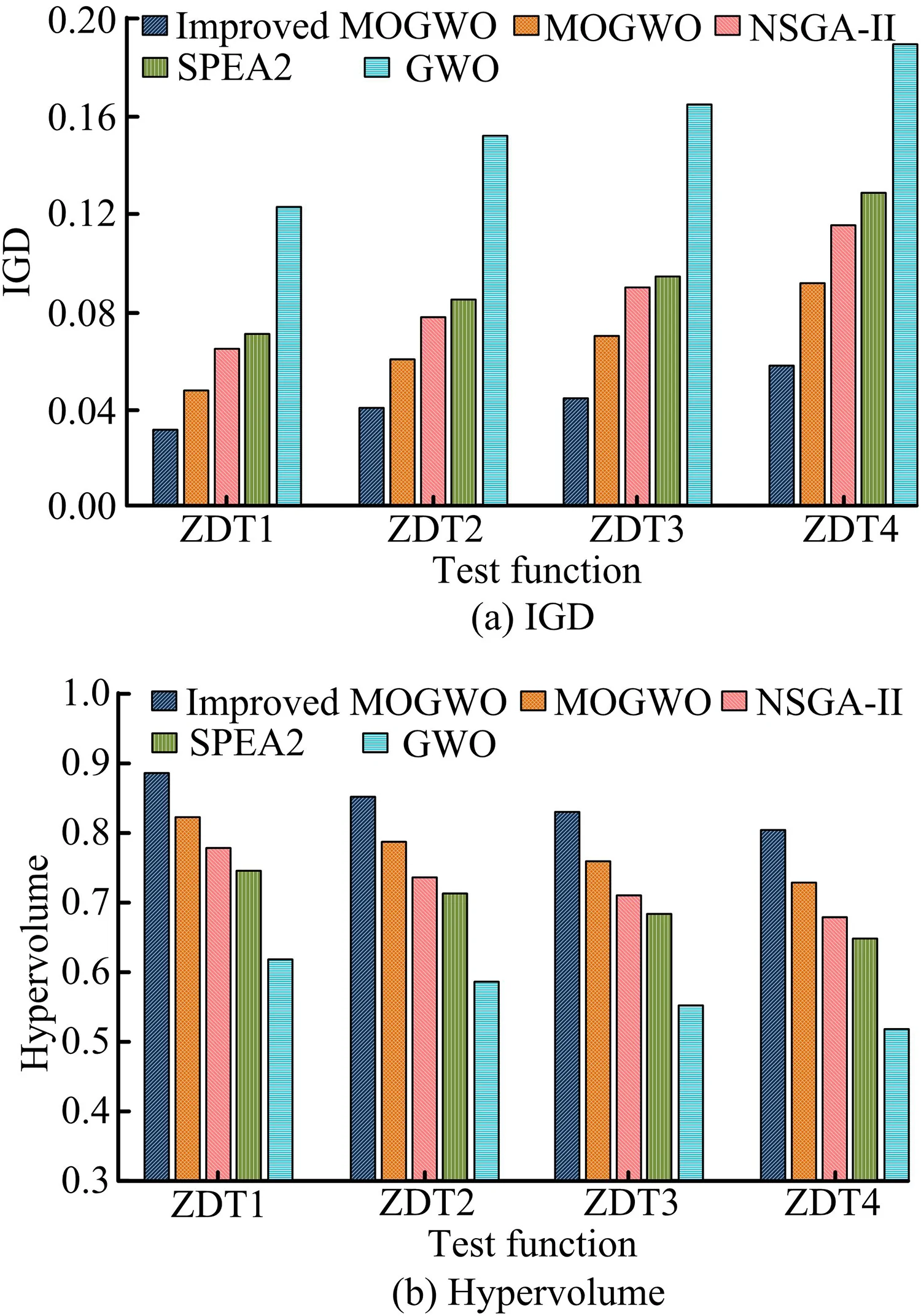
Comparison of IGD and Hypervolume indicators of different algorithms under multi-objective testing function.

To verify the statistical significance of the performance improvement reported by the improved MOGWO algorithm, rather than being caused by random fluctuations, the Wilcoxon signed rank test was performed on all comparison results. This non-parametric test was used to determine whether the median difference between the improved MOGWO and any comparison algorithm is significant on specific test questions and metrics. The study set a significance level of α = 0.05, and tested under high-dimensional testing problems. In addition, each group underwent 30 independent runs. The comparison of indicators for different algorithms is shown in Table 3. Improved MOGWO significantly outperformed other compared algorithms in all test questions and performance metrics. This provides solid statistical evidence for the comprehensive advantages of improved algorithms in terms of convergence (IGD), Hypervolume, and distribution diversity (Spread/GD). In terms of computational cost, GWO had the simplest structure and no multi-objective sorting mechanism, making it the fastest. MOGWO and improved MOGWO were slightly slower than GWO due to the addition of Pareto sorting and adaptive strategies, with the improved individual exchange judgment of MOGWO bringing a small amount of additional overhead. NSGA-II and SPEA2 had significantly higher running times than GWO algorithms due to the use of complex mechanisms such as non-dominated sorting and crowding distance calculation.

**Table 3:** Statistical testing results of algorithms under high-dimensional testing problems.

Index	Testing problems	Improved MOGWO	MOGWO	NSGA-II	SPEA2	GWO
IGD	Door1	0.065 ± 0.013↑	0.105 ± 0.028	0.132 ± 0.038	0.145 ± 0.041	0.211 ± 0.062
	Door2	0.073 ± 0.015↑	0.121 ± 0.032	0.158 ± 0.045	0.172 ± 0.049	0.235 ± 0.071
	Door3	0.082 ± 0.018↑	0.145 ± 0.041	0.198 ± 0.056	0.215 ± 0.063	0.289 ± 0.085
Hypervolume	Door1	0.789 ± 0.035↑	0.702 ± 0.048	0.651 ± 0.056	0.623 ± 0.061	0.485 ± 0.082
	Door2	0.756 ± 0.041↑	0.663 ± 0.055	0.602 ± 0.063	0.578 ± 0.068	0.452 ± 0.091
	Door3	0.721 ± 0.048↑	0.615 ± 0.063	0.552 ± 0.071	0.518 ± 0.078	0.316 ± 0.105
Spread/GD	Door1	0.198 ± 0.022↑	0.305 ± 0.045	0.367 ± 0.051	0.401 ± 0.058	0.523 ± 0.089
	Door2	0.215 ± 0.028↑	0.331 ± 0.049	0.389 ± 0.056	0.425 ± 0.062	0.551 ± 0.094
	Door3	0.241 ± 0.035↑	0.362 ± 0.054	0.421 ± 0.061	0.458 ± 0.067	0.598 ± 0.101
Total running time (ms)	Door1-Door3	185 ± 12	178 ± 11	245 ± 18	238 ± 16	168 ± 10

Note: “ ↑ ” represents the difference between the improved MOGWO and all compared algorithms on this indicator, which has passed the Wilcoxon signed rank test with a significance level of α = 0.05.

To explore the independent contributions of each module and evaluate their synergistic effects, ablation experiments were conducted on Door3 high-dimensional testing problems, and the results are shown in Table 4. Table 4 shows that on the IGD index, introducing the optimal set initialization alone reduces the baseline MOGWO by 18.6%, introducing the adaptive dual population strategy alone reduces it by 33.8%, and combining the two reduces it by 43.4%, demonstrating a significant synergistic enhancement effect. On the Hypervolume metric, the individual contribution of the adaptive dual population strategy (+12.5%) is significantly greater than that of the optimal set initialization (+7.0%), indicating that the dynamic population partitioning strategy plays a dominant role in improving the quality of the solution set. The synchronous improvement of Spread and GD indicators further validates the complementarity of the two mechanisms in maintaining the uniformity of solution set distribution and convergence accuracy. The average convergence generation of the fully improved MOGWO algorithm is reduced by 51.9% compared to the benchmark algorithm, and the standard deviation is reduced to 18 generations, indicating that the improved algorithm not only has a faster convergence speed, but also significantly improves the stability of the convergence process. The improved variants are basically on par with the benchmark algorithm in terms of running time, indicating that the introduced mechanism does not bring significant computational burden. Based on various indicators, the optimal set initialization improves search efficiency by improving the quality of the initial population, while the adaptive dual population strategy avoids premature convergence by dynamically balancing local development and global exploration. The synergistic effect of the two approaches achieves optimal results in all evaluation dimensions.

**Table 4:** Results of ablation experiment.

Algorithm variants	IGD	Hypervolume	Spread	GD	Convergent algebra	Running time (s)
Benchmark MOGWO	0.145 ± 0.041	0.615 ± 0.063	0.421 ± 0.061	0.198 ± 0.056	412 ± 38	178 ± 11
MOGWO+Optimal Set Initialization	0.118 ± 0.032	0.658 ± 0.055	0.367 ± 0.052	0.162 ± 0.048	356 ± 32	181 ± 12
MOGWO+adaptive dual population strategy	0.096 ± 0.025	0.692 ± 0.051	0.312 ± 0.044	0.128 ± 0.039	278 ± 25	183 ± 12
Fully Improved MOGWO	0.082 ± 0.018	0.721 ± 0.048	0.241 ± 0.035	0.089 ± 0.028	198 ± 18	185 ± 12

### 3.3. Solution and analysis of optimization model for car door structure

To evaluate the effectiveness of the proposed hierarchical optimization model and the improved MOGWO algorithm, a benchmark car door initial design was used as the starting point for optimization and used as the baseline for comparison. Subsequently, the optimization framework proposed in this study was applied to perform MOO on the initial design, aiming to synergistically enhance its aesthetics and safety. The experiment optimized the car door structure by improving the MOGWO algorithm. The pre – and post optimization values of key parameters in the geometric layer, structural layer, and material layer are shown in Fig 9. In the geometric layer of Fig 9(a), the curvature radius of the outer plate was optimized from 1500 mm to 1800mm, and the inclination angle of the characteristic line was adjusted from 25 ° to 22 °, improving the uniformity of the surface. As shown in Fig 9(b), the width and height of the anti-collision beam section of the structural layer were increased by 10 mm and 8 mm respectively, and the length of the energy absorbing box was extended from 120 mm to 150 mm, enhancing the collision energy absorption capacity. In Fig 9(c), the material layer used a plate with a tensile strength of 500MPa and an elongation of 25%, achieving a better balance between strength and plasticity compared to the original parameters (400MPa, 20%).

**Fig 9:**
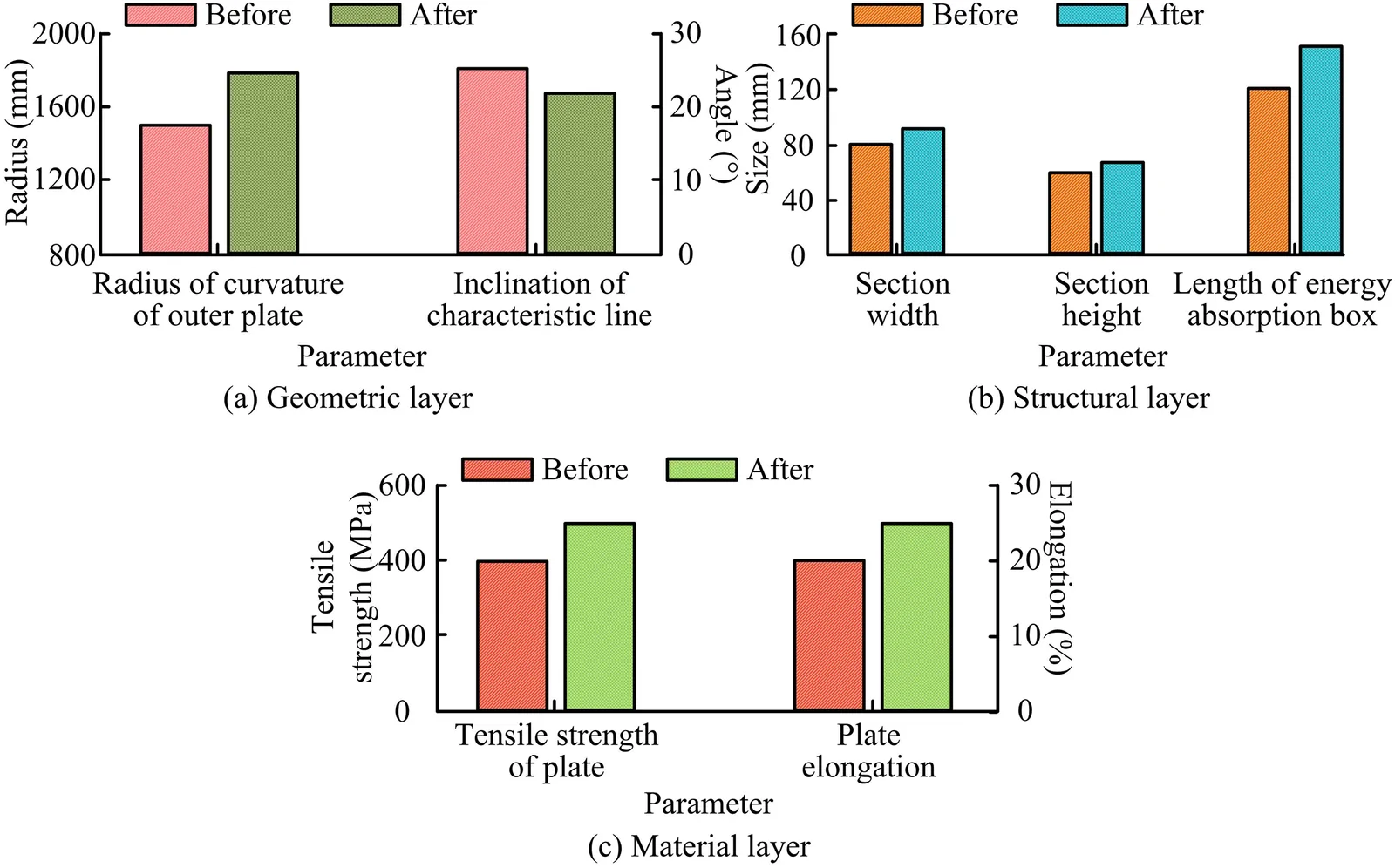
Pre – and post optimization values of key parameters for geometric, structural, and material layers.

The comparison of aesthetics and safety before and after optimization is shown in Fig 10. In Fig 10(a), the aesthetic function value increased from 0.62 before optimization to 0.89 after optimization, indicating a significant improvement in the uniformity of the outer panel curvature and a smoother visual effect. As shown in Fig 10(b), the collision intrusion amount decreased from 65 mm before optimization to 42 mm after optimization. In Fig 10(c), the chest acceleration decreased from 45g before optimization to 32g after optimization. Both the collision intrusion amount and chest acceleration exceeded the safety standard limits. In Fig 10 (d), the safety function value decreased from 0.75 before optimization to 0.32 after optimization, reflecting a significant reduction in the weighted risk of collision intrusion and chest acceleration, and an improvement in safety.

**Fig 10:**
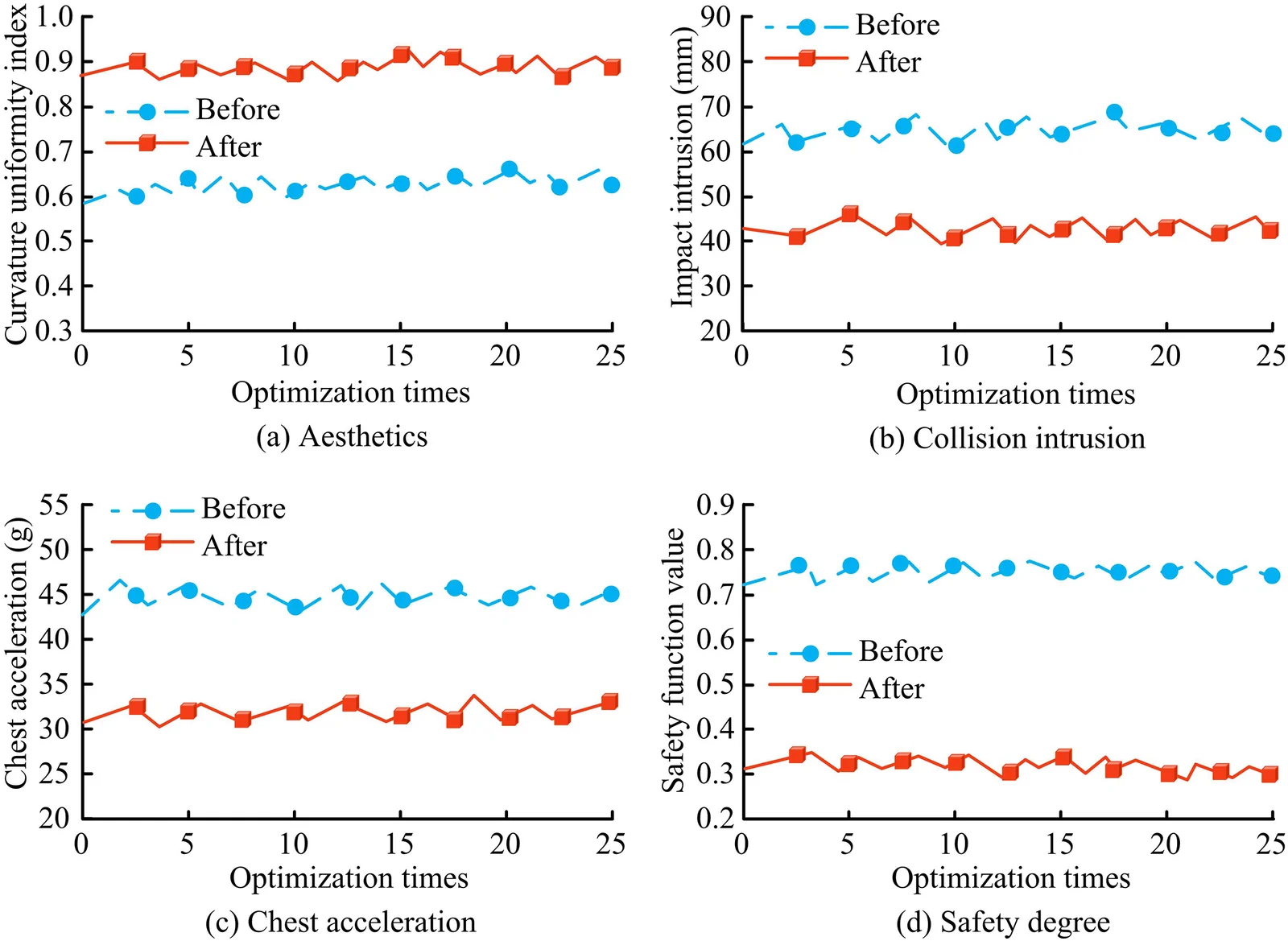
Comparison of aesthetics and safety before and after optimization.

To further investigate the impact of aesthetic and safety weights on optimization results, three additional weight combinations were set up in the experiment. The impact of aesthetic and safety weights on optimization results is shown in Table 5. When the aesthetic weight increased to 0.7, the curvature radius of the outer panel increased to 2000mm, the inclination angle of the characteristic line further decreased to 20 °, and the CUI increased to 0.92. However, the growth rate of the anti-collision beam section size decreased, and the safety function value slightly increased to 0.35. When the safety weight was 0.7, the length of the energy absorbing box was extended to 160 mm, and the collision intrusion was reduced to 38 mm, sacrificing some aesthetics for higher safety. This indicates that designers can flexibly adjust weights and obtain customized solutions based on vehicle positioning (such as emphasizing safety for home use and aesthetics for sports cars).

**Table 5:** The impact of aesthetic and safety weights on optimization effectiveness.

Index	$w_{1}:w_{2}$		
	0.3:0.7	0.5:0.5	0.7:0.3
Outer plate curvature (mm)	1700	1800	2000
Characteristic line inclination angle (°)	23	22	20
Anti-collision beam width (mm)	95	90	85
Length of the energy-absorbing box (mm)	160	150	140
*CUI*	0.85	0.89	0.92
*S*	0.28	0.32	0.35

To ensure the reliability of finite element simulation results and provide effective performance benchmarks for subsequent optimization, the collision simulation results of the benchmark car door model are compared and verified with the reference model provided by the original equipment manufacturer. The reference model is established based on the standard door structure of the same model, and its geometric parameters, material properties, and boundary conditions have been calibrated through physical experiments, which can serve as a reliable benchmark for comparison and verification. Under the same computational conditions, two models were run six times each to evaluate the numerical stability of the simulation results. Five indicators, including maximum intrusion, residual deformation, chest acceleration, peak intrusion time, and total energy absorption, were selected for comparative analysis. Paired t-test is used to determine whether there are statistically significant differences between the benchmark model and the reference model in various indicators, with a significance level of α = 0.05. The results are shown in Table 6. Table 6 shows that the absolute deviation between the benchmark model and the reference model in five indicators is less than 5%. The paired t-test results showed that the *p*-values of all indicators were greater than 0.05, indicating no statistically significant difference between the two. The deviation of chest acceleration is 3.0%, slightly higher than other indicators, which may be related to differences in local parameter settings at the contact interface of the dummy model. The small standard deviation of each indicator reflects the good numerical stability of the simulation solution process. The above verification results confirm that the benchmark car door model has good consistency with the reference model in terms of the main collision response characteristics, meets the requirements of engineering simulation accuracy, and can be used as a reliable benchmark for subsequent structural optimization analysis.

**Table 6:** Comparison of verification results between benchmark door model and reference model.

Validation metrics	Reference model	Baseline model	Deviation (%)	T-value	*p*-value
Maximum intrusion amount (mm)	62.3 ± 2.1	63.8 ± 2.4	2.4	1.82	0.128
Intrusion residual deformation (mm)	28.5 ± 1.8	29.2 ± 2.0	2.5	1.06	0.337
Chest acceleration (g)	43.2 ± 1.5	44.5 ± 1.9	3	2.31	0.069
Peak intrusion time (ms)	68.5 ± 2.3	67.2 ± 2.6	−1.9	−1.55	0.182
Total energy absorption (kJ)	8.42 ± 0.31	8.28 ± 0.35	−1.7	−1.22	0.276

The optimized collision performance was verified through finite element simulation, and the intrusion and acceleration data of key nodes are denoted in Fig 11. Fig 11(a) showcases the intrusion test results. The intrusion in the middle of the car door was 58 mm before optimization and decreased to 39 mm after optimization. The intrusion at the B-pillar connection was 45 mm before optimization and decreased to 32 mm after optimization. Both optimized values were below the safety threshold (≤ 50 mm). The acceleration test results in Fig 11(b) showed that the collision acceleration in the middle of the car door was 48g before optimization and decreased to 34g after optimization. The acceleration at the B-pillar connection was 52g before optimization and decreased to 36g after optimization, both of which meet the safety threshold (≤ 35g) requirements, demonstrating the significant effectiveness of optimization measures in reducing intrusion and acceleration.

**Fig 11:**
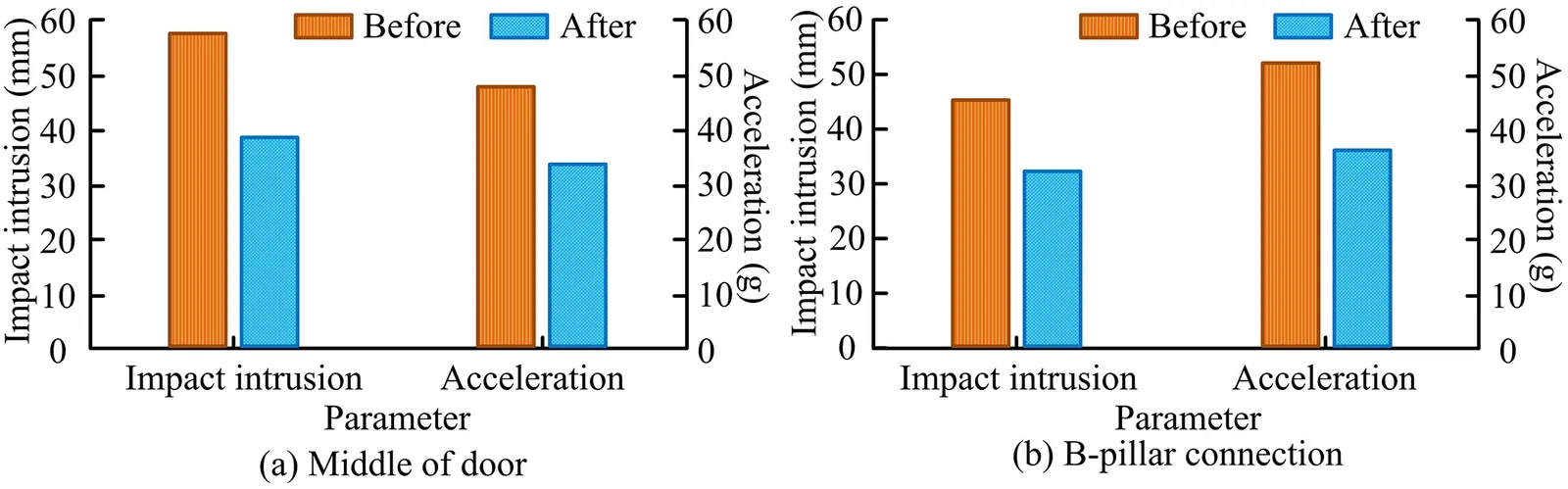
Intrusion and acceleration data of key nodes.

The optimization effect of material parameters is shown in Table 7. An increase of 25% in tensile strength increased the overall stiffness of the car door by 18%, and improved its anti dent performance by 22%, meeting the requirements of aesthetics for surface stiffness. An increase of 5% in elongation enhanced the plastic deformation ability of the material before fracture, and the energy absorbing box could produce greater plastic deformation during collision. With the optimization of structural parameters, the overall energy absorption efficiency was improved by 17%. In addition, the material density remained unchanged at 7.85g/cm^3^, and the optimized door mass only increases by 1.2 kg, controlling weight growth while improving performance.

**Table 7:** Optimization effect of material parameters.

Material parameters	Before optimization	After optimization
Tensile strength (MPa)	400	500
Elongation (%)	20	25
Elastic modulus (GPa)	210	210
Density (g/cm^3^)	7.85	7.85

## 4. Discussion

The research has achieved practical results by improving the MOGWO algorithm and applying it to optimize the structure of car doors. On the ZDT series test functions, the improved MOGWO algorithm showed lower IGD values and higher Hypervolume values compared to the original MOGWO and other comparative algorithms. This advantage can be attributed to the adaptive dual population strategy, which dynamically adjusts the ratio of local exploitation to global exploration, effectively avoiding the algorithm from getting stuck in local optima and improving the stability of the convergence process. In the optimization of car door structure, the improvement of aesthetic function value and the decrease of collision intrusion and chest acceleration after optimization indicate that the combination of the proposed layered parametric model and the improved MOGWO algorithm can achieve collaborative optimization between aesthetics and safety. The improvement of curvature uniformity is related to the adjustment of the curvature radius and characteristic line inclination of the outer plate, while the improvement of collision performance is mainly due to the increase in the cross-sectional size of the collision beam and the extension of the length of the energy absorbing box. In addition, the advantages of the improved algorithm in the Hypervolume metric indicate that the synergistic effect of optimal set initialization and dual population strategy not only accelerates convergence speed, but also maintains the wide distribution of Pareto front in the target space, providing designers with more diverse non dominated solution sets. Traditional GWO is prone to getting stuck in local optima, while MOGWO improves multi-objective processing capabilities but lacks population diversity. However, research has found that by initializing the best point set, the initial population covered a more comprehensive search space, while the dual population strategy dynamically maintained a balance between exploration and development during iterations. This is consistent with the conclusion in the literature that adaptive strategies can improve algorithm robustness [31]. In the optimization of car doors, the layered parameter model integrates geometric, structural, and material parameter systems, and achieves a quantitative balance between aesthetics and safety through multi-objective functions, which compensates for the limitations of single performance optimization in existing research.

The optimization framework proposed in this study is primarily validated within the context of automotive door design. Its methodological structure, which integrates algorithmic improvement, hierarchical parameter modeling, and multi-objective evaluation, may be adaptable to other engineering design problems that involve similar conflicts between morphological characteristics and functional performance. At the algorithmic level, the improved MOGWO algorithm effectively addresses the issues of insufficient population diversity and local optimal traps through optimal set initialization and adaptive dual population strategies. This mechanism is universal for any complex optimization problem with high-dimensional, nonlinear and objective conflict characteristics. For instance, in the aerospace field, this algorithm can be applied to the collaborative design of the aerodynamic shape and structural strength of wings. In the fields of architecture and civil engineering, it can be used for MOO of building appearance aesthetics and structural seismic performance. At the modeling level, the constructed “geometric-structure-material” hierarchical parameter model provides a clear approach for dealing with multi-physics and multi-disciplinary design problems. This method of logically stratifying parameters from different fields based on their physical nature and design intent can be transferred to other complex product designs. For instance, in the development of consumer electronic products, a hierarchical optimization model covering the appearance shape (geometric layer), internal support and heat dissipation structure (structural layer), and shell material (material layer) can also be established to balance aesthetics, durability, and heat dissipation efficiency. At the evaluation system level, the subjective aesthetic quality is quantified through indicators such as CUI, and combined with objective “safety” performance indicators to construct a multi-objective function, providing a referential idea for handling other engineering design problems involving subjective preferences.

However, the research still has certain limitations. One is the sensitivity of the algorithm parameters. The sensitivity of these parameters in different types of optimization problems has not been systematically studied, which may affect the robustness of the algorithm in new domains without parameter tuning. The second is the completeness of the aesthetic evaluation model. The current aesthetic function is only based on the uniformity of curvature, which, although an important visual feature, does not fully cover users’ complex subjective aesthetic preferences, such as the sharpness, fullness or family characteristics of the shape. The aesthetic evaluation model still needs to incorporate more visual cognitive factors and psychological principles to enhance its consistency with human subjective evaluation. The third is the singularity of the security verification scenario. The simulation verification only focuses on the performance of key nodes in side collisions and has not yet been extended to more complex collision scenarios, such as offset collisions at different angles, column collisions, and collision scenarios at various speeds. A more comprehensive multi-scenario verification system is crucial for ensuring the robustness of the optimization scheme in actual road environments.

Therefore, in future further research, it can be carried out from the following directions. The first is to develop an adaptive parameter adjustment mechanism to reduce the reliance on manual empirical parameter adjustment and enhance the algorithm’s adaptive ability and universality. The second is to explore the integration of subjective evaluation data such as eye movement experiments and user surveys with advanced technologies like deep learning to construct a data-driven aesthetic evaluation model that better reflects the complex aesthetic consensus of humanity. Thirdly, the optimization framework is combined with a high-precision vehicle collision simulation model to carry out systematic safety verification covering various regulations and measured working conditions, ensuring the reliability of the optimized design scheme under all working conditions.

## 5. Conclusion

For the MOO problem of automobile door structure, this study proposed an improved MOGWO algorithm and a hierarchical optimization model. In terms of algorithm improvement, the population distribution uniformity was improved by initializing the best point set, and the adaptive dual population strategy was used to dynamically adjust the ratio of local development and global exploration, effectively solving the problems of traditional MOGWO being prone to falling into local optima and insufficient convergence stability. In terms of optimizing the model, the constructed geometric structural material layered parameter model clearly outlined the key parameters of the car door, and based on the CUI and weighted risk value multi-objective function, achieved quantitative optimization of aesthetics and safety. Experiments showed that the improved algorithm reduced the IGD value by 32.7% compared to the original MOGWO and increased the Hypervolume value by 24.5% compared to SPEA2 in the ZDT series test functions. Its advantages were more significant in high-dimensional problems, verifying its superior optimization performance. The optimization results showed that the CUI of the car door was increased from 0.62 to 0.89, the collision intrusion was reduced from 65 mm to 42 mm, the chest acceleration was reduced from 45g to 32g, and the weight adjustment could meet the customized needs of different car models. In summary, this study integrates an improved MOGWO algorithm with a hierarchical parametric model for car door multi-objective optimization. Experimental results show measurable gains in both aesthetic and safety indicators for the tested problem. The adaptive dual-population strategy enhances convergence and solution diversity on evaluated functions and tasks. The hierarchical model systematically represents key design variables across geometric, structural, and material layers. The multi-objective function enables quantitative trade-off assessment between aesthetics and collision safety within the defined framework.
